# MiR-199a Regulates Cell Proliferation and Survival by Targeting FZD7

**DOI:** 10.1371/journal.pone.0110074

**Published:** 2014-10-14

**Authors:** Jiugang Song, Liucun Gao, Guang Yang, Shanhong Tang, Huahong Xie, Yongji Wang, Jingbo Wang, Yanping Zhang, Jiang Jin, Yawen Gou, Zhiping Yang, Zheng Chen, Kaichun Wu, Jie Liu, Daiming Fan

**Affiliations:** 1 Department of Gastroenterology, the 309th Hospital of Chinese People's Liberation Army, Beijing, PR China; 2 State Key Laboratory of Cancer Biology, Xijing Hospital of Digestive Diseases, Fourth Military Medical University, Xi'an, PR China; 3 Department of Pharmacology and Toxicology, Beijing Institute of Radiation Medicine, Beijing, PR China; 4 Department of XiShan Outpatient Clinic, the 309th Hospital of Chinese People's Liberation Army, Beijing, PR China; 5 Department of Digestion, General Hospital of Chengdu Military Command, Chengdu, Sichuan Province, PR China; 6 Department of Medical, the 309th Hospital of Chinese People's Liberation Army, Beijing, PR China; 7 Asian Games Village Clinic of Logistics Department, the General Armament Department of PLA, Beijing, PR China; 8 Department of Digestive Diseases, Huashan Hospital, Fudan University, Shanghai, PR China; The University of Hong Kong, China

## Abstract

A growing amount of evidence indicates that miRNAs are important regulators of multiple cellular processes and, when expressed aberrantly in different types of cancer such as hepatocellular carcinoma (HCC), play significant roles in tumorigenesis and progression. Aberrant expression of miR-199a-5p (also called miR-199a) was found to contribute to carcinogenesis in different types of cancer, including HCC. However, the precise molecular mechanism is not yet fully understood. The present study showed that miR-199a is frequently down-regulated in HCC tissues and cells. Importantly, lower expression of miR-199a was significantly correlated with the malignant potential and poor prognosis of HCC, and restoration of miR-199a in HCC cells led to inhibition of the cell proliferation and cell cycle *in vitro* and *in vivo*. Furthermore, Frizzled type 7 receptor (FZD7), the most important Wnt receptor involved in cancer development and progression, was identified as a functional target of miR-199a. In addition, these findings were further strengthened by results showing that expression of FZD7 was inversely correlated with miR-199a in both HCC tissues and cells and that over-expression of miR-199a could significantly down-regulate the expression of genes downstream of FZD7, including β-catenin, Jun, Cyclin D1 and Myc. In conclusion, these findings not only help us to better elucidate the molecular mechanisms of hepatocarcinogenesis from a fresh perspective but also provide a new theoretical basis to further investigate miR-199a as a potential biomarker and a promising approach for HCC treatment.

## Introduction

Hepatocellular carcinoma (HCC), the most common primary liver cancer [1], is one of the most prevalent malignant diseases and the second-most frequent cause of cancer deaths worldwide [2]. Half of the new liver cancer cases and liver cancer deaths worldwide were estimated to occur in China [3]. The dismal prognosis of advanced HCC is largely caused by late detection of the tumors and its high rate of recurrence and metastasis [2], [4], [5]. Etiologically, approximately 90% of HCC cases arise from cirrhosis, and the disease is strongly associated with several risk factors, including viral infections (e.g., hepatitis B and C) and heavy alcohol intake [6].

In the past decades, studies have focused on investigating the deregulation of genes and proteins underlying the development of HCC [7]. MiRNAs are a recently discovered class of small noncoding RNAs that play critical roles in regulating gene expression. MiRNAs have emerged as key factors involved in several biological processes, including development, differentiation, cell proliferation, and tumorigenesis [8], [9]. Several studies have shown that alterations in miRNA genes lead to tumor formation, and several miRNAs that regulate either tumor suppression or tumor formation have been identified [10].

Recently, an increasing number of studies have demonstrated that the expression of miRNAs is deregulated in HCC in comparison with normal liver tissue [11], [12]. In view of reports from independent studies, consistent deregulation of miR-21, miR-122, miR-199, and miR-221 appears to be particularly important in HCC [13], [14], [15], [16], [17], [18]. Interestingly, both miR-122 and miR-199a are among the miRNAs that are most highly expressed in normal liver [19].

However, the role and underlying molecular mechanisms of miR-199a in HCC is not completely understood. The present study aimed to analyze the expression of miR-199a in HCC tissues compared with adjacent non-tumor tissues and to analyze its role in the malignant progression of HCC *in vitro* and *in vivo*. In addition, bioinformatics predicted that FZD7, the most important Wnt receptor, might be a target of miR-199a. To further test this hypothesis, we analyzed the influence of miR-199a on FZD7 and on the expression of its downstream genes.

## Materials and Methods

### Cell Lines and Human Samples

Human HCC cells (SMMC7721 and HepG2) and normal hepatocytes (Chang liver cells) were obtained from the Cell Research Institute of the Chinese Academy of Sciences (Shanghai, China). 293FT cells were obtained from the American Type Culture Collection (ATCC, Manassas, VA). All of the cell lines were cultured in Dulbecco's modified Eagle medium (Gibco BRL, Life Technologies, NY) supplemented with 10% fetal calf serum (FCS), 100 µg/ml penicillin, and 100 µg/ml streptomycin at 37°C in a 5% CO_2_ incubator. HCC specimens and paired non-tumor liver tissues were obtained from 40 patients who underwent primary HCC resection between June 2006 and January 2007 at the Department of General Surgery in Xijing Hospital (Xi'an, China). No patient had received radiotherapy or chemotherapy before surgery. Data including sex, age, tumor size, histologic type of neoplasm and tumor-node-metastasis (TNM) stage were obtained from surgical and pathological records, and all samples were thoroughly reviewed by two pathologists. Tissue samples were collected at surgery, immediately snap-frozen in liquid nitrogen and stored at −80°C until RNA extraction. Written informed consent was obtained from all patients and the study was approved by the Ethics Committee of Xijing Hospital, Fourth Military Medical University, China.

### RNA Extraction and Real Time-PCR Analysis (qRT-PCR)

In accordance with the manufacturer's instructions, total RNA was extracted from the cell lines and frozen tissue specimens with TRIzol reagent (Invitrogen, Carlsbad, CA, USA), and the concentration of the total RNA was quantitated by measuring the absorbance at 260 nm. Complementary DNA (cDNA) was generated using a miScript Reverse Transcription Kit (QIAGEN), and Real-time PCR was performed using a miScript SYBRGreen PCR Kit from QIAGEN. Primers for miR-199a and the U6 snRNA (internal control) were also purchased from QIAGEN (MS00006741 and MS00033740). The fold-change of the mRNA in HCC tissues (T) relative to the adjacent non-tumor tissues (NT) was calculated using a previously described method [20], where ΔΔCt = ΔCt(T)-ΔCt(NT) and ΔCt = Ct(miR-199a)-Ct(U6). The relative mRNA levels of genes in HCC cells were also determined using qRT-PCR, and the expression of GAPDH was used as the internal control. Each PCR was performed in triplicate. The primers for the examined genes are presented in Table 1.

**Table 1:** Primers for qRT-PCR.

Primers	Sequence
FZD7	F 5′ TTTCGTCCCTGGGCCTCT 3′
	R5′ TGGTCTGGTTGTAGGCGATG3′
GAPDH	F5′ GCACCGTCAAGGCTGAGAAC 3′
	R 5′ TGGTGAAGACGCCAGTGGA 3′

### Vector Constructs and Lentivirus Production

Lentivirus-expressed miR-199a was constructed as described in a previous study [21]. The precursor sequence of miR-199a was constructed as follows: (Forward) hsa-miR-199a-Xho GGGCCCGCTCTAGACTCGAGATATTTGCATGTCGCTATGTG, (Reverse) hsa-miR-199a- BamH I CGCGGCCGCCTAATGGATCCAAAAAAGGCACAGTCGAGGCTGATC. The sequence was amplified and cloned into the pGCSIL-GFP Vector (GENECHEM) to generate pGCSIL-GFP-miR-199a. The miR-199a and the negative-control virus were transfected into HepG2 cells as in a previous study [21]. Cells successfully transfected with GFP were separated by flow cytometry (FACScan; Becton Dickinson, San Jose, CA), and the purified, GFP-positive cell lines were named HepG2-199a and HepG2-NC (NC, negative control).

### Cell Proliferation Assays

An MTT assay was used to analyze cellular proliferation according to a previously described protocol [22]. Briefly, log-phase cells were plated in 96-well plates (1×10^3^ cells/well at a final volume of 200 µl) in replicates of three. After 1, 2, 3, 4, 5, 6 or 7 days of cell cultivation, 20 µl of 3-(4,5-dimethylthiazol-2-yl)-2,5-diphenyltetrazolium bromide (MTT, 5 mg/ml; Sigma, St. Louis, MO) was added to the cells and incubated for 4 hours at 37°C. The supernatant was then removed, and 150 µl dimethylsulfoxide (DMSO) was added with agitation for 10 minutes at room temperature to dissolve the MTT crystals. The absorbance values were determined by an ELISA reader (Bio-Rad Laboratories, Richmond, CA) at a wavelength of 490 nm. Each experiment was repeated at least thrice.

### Soft Agar Assay

The tumorigenicity of the cells *in vitro* was determined by analyzing the formation of colonies in soft agar. Approximately 2×10^3^ cells were seeded in 2 ml of 0.3% agar layered onto a 0.5% agar underlay in a six-well plate. Cells were incubated for 3 weeks at 37°C in 5% CO_2_ before counting the colonies using a code. Each assay was performed in triplicate.

### Flow Cytometry Assay

For cell cycle analysis, cells were harvested and washed twice with ice-cold phosphate-buffered saline (PBS). The cell pellets were fixed in 70% ethanol, treated with RNase A (Boehringer Mannheim, Indianapolis, IN), and stained with propidium iodide (Sigma-Aldrich, St. Louis, MO). Cell cycle analysis was performed with a flow cytometer (FACScan; Becton Dickinson, San Jose, CA). The proliferation index (PI) was calculated as PI = (S+G2)/(S+G2+G1).

### Luciferase Reporter Assay

For dual luciferase reporter assays, a luciferase reporter vector (pMir-Report; Ambion) was used to generate luciferase reporter constructs. A fragment of the 3′-UTR of the FZD7 mRNA (region 1974–2508, GenBank accession no. NM_003507), which included the seed sequence of the mature miR-199a-binding site, and a mutated binding site of the 3′-UTR sequence were cloned into the luciferase reporter vector. HepG2-199a or HepG2-NC cells in 24-well plates were co-transfected with 0.2 µg of the firefly luciferase reporter vector and 0.08 µg of the pRL-TK control vector containing Renilla luciferase (Promega) using Lipofectamine 2000 (Invitrogen) according to the manufacturer's protocol. Lysates were prepared after 48 h of transfection. Both firefly luciferase and Renilla luciferase activities were measured using the Dual-Luciferase assay kit (Promega, Madison, WI) according to the manufacturer's instructions. Firefly luciferase activity was normalized to Renilla luciferase activity for each transfected well. Three independent experiments were performed in triplicate. The primers for the 3′-UTR of FZD7 and mutated 3′-UTR of FZD7 sequences are shown in Table 2.

**Table 2:** Primers for the 3′UTR of FZD7 and Mutant-FZD7.

Primers	Sequence
FZD7	F 5′ CTAG ACTAGT GAAAGCGGTTTGGATGAA 3′
	R 5′ CCC AAGCTT CGTCTCCTTGGCCTTATC 3′
Mutant-FZD7	F 5′ CG ACGCGT GCCAAACTGGAGCCCAGAT 3′
	R 5′ CG ACGCGT AAAAAGAGATTATGGTTTGA 3′

### Western Blot Analysis

Western Blot analysis was performed according to our previous study [22]. Total cellular proteins were extracted and separated using sodium dodecyl sulfate–polyacrylamide gel electrophoresis and transferred to nitrocellulose membranes (Immobilin-P; Millipore, Bedford, MA). The membranes were blocked with 5% nonfat milk at room temperature for 2 hours and then incubated overnight with rabbit anti-FZD7 (Santa Cruz, CA, USA), mouse anti-Myc (Santa Cruz, CA, USA), mouse anti-Cyclin D1 (Santa Cruz, CA, USA), mouse anti-β-catenin (Santa Cruz, CA, USA) or mouse anti-β-actin (Sigma, St. Louis, MO, USA) antibodies at 4°C. After incubation with horseradish peroxidase–conjugated anti-rabbit IgG or anti-mouse IgG (Santa Cruz, CA, USA), the specific protein band was visualized by enhanced chemiluminescence (Amersham-Pharmacia Biotech, Beijing, China). β-actin was used as an internal control, and each experiment was repeated at least thrice.

### Tumorigenicity Assays in Nude Mice

Female athymic BALB/c nude mice (4–5 weeks old) were purchased from the Animal Center of the Chinese Academy of Science (Shanghai, China), maintained in laminar flow cabinets under specific pathogen-free conditions, and had free access to food and water. All animal studies were undertaken in accordance with the National Institutes of Health Guide for the Care and Use of Laboratory Animals and approved by the Institutional Animal Care and Use Committee (IACUC) of the Fourth Military Medical University (Permit Number: 12020). Twelve mice were assigned to 2 groups and used for *in vivo* tumorigenicity assays. Logarithmically growing cells were trypsinized and resuspended in PBS after washing twice with serum-free medium. HepG2-199a and HepG2-NC cells (2×10^6^) were injected subcutaneously into the flanks. Four weeks after inoculation, the tumor-bearing mice were euthanized to recover the tumors for further analysis, and all efforts were made to minimize suffering. Tumor volume was measured using a Vernier caliper, and tumor volumes were calculated following the formula [22]: Tumor volume (cm^3^) = (a/2)(b/2)hπ, where a, b, and h are the minor dimension, major dimension, and height of the tumor, respectively (π = 3.1416).

### Statistical Analysis

All data were analyzed using the SPSS software package (SPSS, Chicago, IL), and *P*<0.05 was considered statistically significant. The significant differences in the expression of miR-199a in the HCC and paired noncancerous tissues were analyzed by the Wilcoxon rank sum test. The Kruskal–Wallis *H* test or the Mann–Whitney *U* test was utilized to evaluate the significance of the correlation between the miR-199a expression and the clinical features of HCC. A one-way ANOVA test was adopted to investigate the difference in cell proliferation and soft agar clonogenic assays of three groups, and the least significant difference T test was used to analyze two groups. Overall survival curves were plotted using the Kaplan-Meier method and were evaluated for statistical significance using a log-rank test.

## Results

### The Expression of miR-199a is Frequently Down-regulated in Human HCC Tissues and Cell Lines

**Figure 1:**
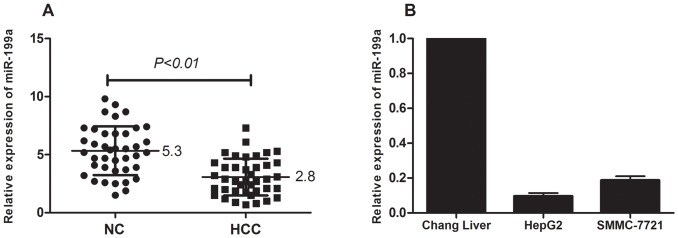
Down-regulation of miR-199a in HCC tissues and cell lines was detected by qRT-PCR. (A) The expression level of miR-199a was lower in 40 HCC tissues than in their pair-matched adjacent normal liver tissues (*P*<0.01). Each sample was analyzed in triplicate and normalized to the U6 snRNA. (B) The relative miR-199a expression in HCC cell lines was much lower compared to the normal Chang liver cell line. The relative expression of miR-199a was normalized to the endogenous control U6 snRNA. Each sample was analyzed in triplicate.

To further determine whether miR-199a is involved in the regulation of tumorigenesis of human HCC, the expression level of miR-199a in HCC and matched nonneoplastic liver tissues from 40 patients was analyzed by qRT-PCR. The results showed that the expression of miR-199a was decreased in 82.5% (33/40) of HCC tissues compared with matched nonneoplastic liver tissues, with an average of 4.79-fold reduction in expression (median  = 2.8 vs 5.3; *P*<0.01; Figure 1A).

The expression of miR-199a in the cell lines displayed a pattern similar to that in the tissues. As shown in Figure 1B, a lower expression of miR-199a was detected in the HCC cell lines HepG2 and SMMC-7721, whereas the expression of miR-199a was higher in the normal Chang liver cell line. MiR-199a expression in HepG2 cells was lower than in SMMC-7721 cells. Therefore, we chose the HepG2 cell lines for further study.

### A Lower miR-199a Expression Level in HCC Tissue Correlates with Worse Prognosis of HCC Patients

**Figure 2:**
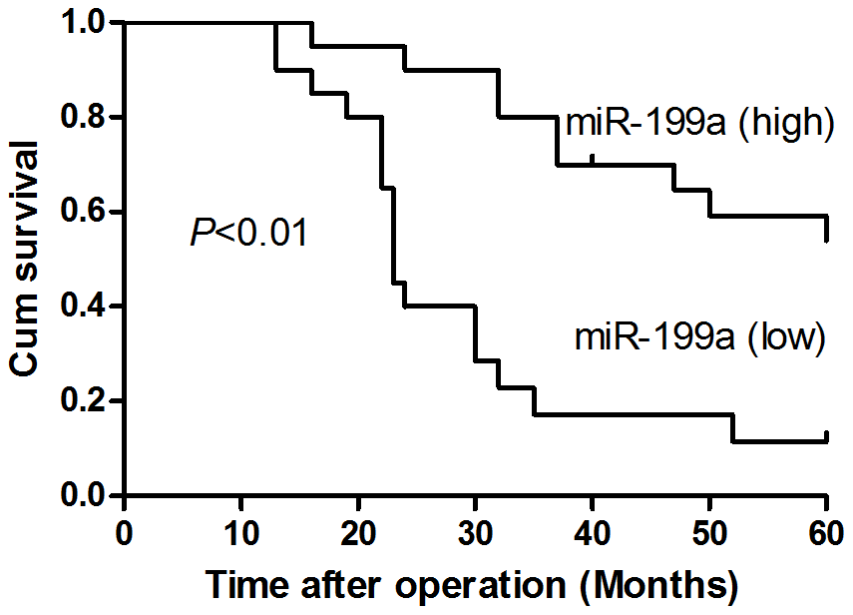
Lower miR-199a expression in HCC tissues is correlated with poorer survival of HCC patients. Five-year cumulative survivals of the HCC patients were analyzed by Kaplan-Meier survival analysis and log-rank tests. The 40 HCC patients were split into high and low miR-199a expression groups based on the median (2.8) for miR-199a. The relative expression of miR-199a in each patient was shown in the supporting information (Table S1).

The correlations between miR-199a expression in HCC tissue and clinical features or prognosis of HCC patients were also studied. Further analysis of the clinical features of 40 HCC patients revealed that the low expression of miR-199a was positively associated with the patients' TNM stage and tumor metastasis (Table 3). Then, the miR-199a expression levels in HCC samples were used for a survival analysis, and the results revealed that down-regulation of miR-199a expression was significantly associated with poor patient prognosis. The median survival time of these patients was 37 months after operation. A Kaplan-Meier analysis revealed that low miR-199a expression was significantly associated with poor 5-year overall survival time (29.2±3.2 months vs 51.8±3.1 months, P<0.001) (Figure 2). Univariate analysis showed that the patient survival time was also significantly correlated with clinical features such as metastasis (P<0.001) and T stage (P = 0.002), but not with age or gender (Table 4).

**Table 3:** MiR-199a expression is correlated with the clinical features of patients.

		MiR-199a		
Parameter	Group	Low	High	*P* value
Age	<52	12	10	0.6
	>52	8	10	
Gender	Men	12	9	0.42
	Women	8	11	
Metastasis	With	4	14	0.01*
	Without	16	6	
T stage	I+II	7	15	0.03*
	III+IV	13	5	

*P<0.05 was considered statistically significant.

**Table 4:** Univariate analysis of clinical parameters associated with prognosis.

Parameter	Chi-square	P value
Age	0.21	0.65
Gender	1.78	0.18
Metastasis	13.8	<0.01*
T stage	9. 8	0.002*
miR-199a	15.1	<0.01*

The univariate analysis showed that metastasis, T stage, and miR-199a were statistically significant prognostic factors for HCC patients.

### Over-expression of MiR-199a Represses the Growth and Tumorigenicity of HCC Cells *In Vitro* and *In Vivo*

**Figure 3:**
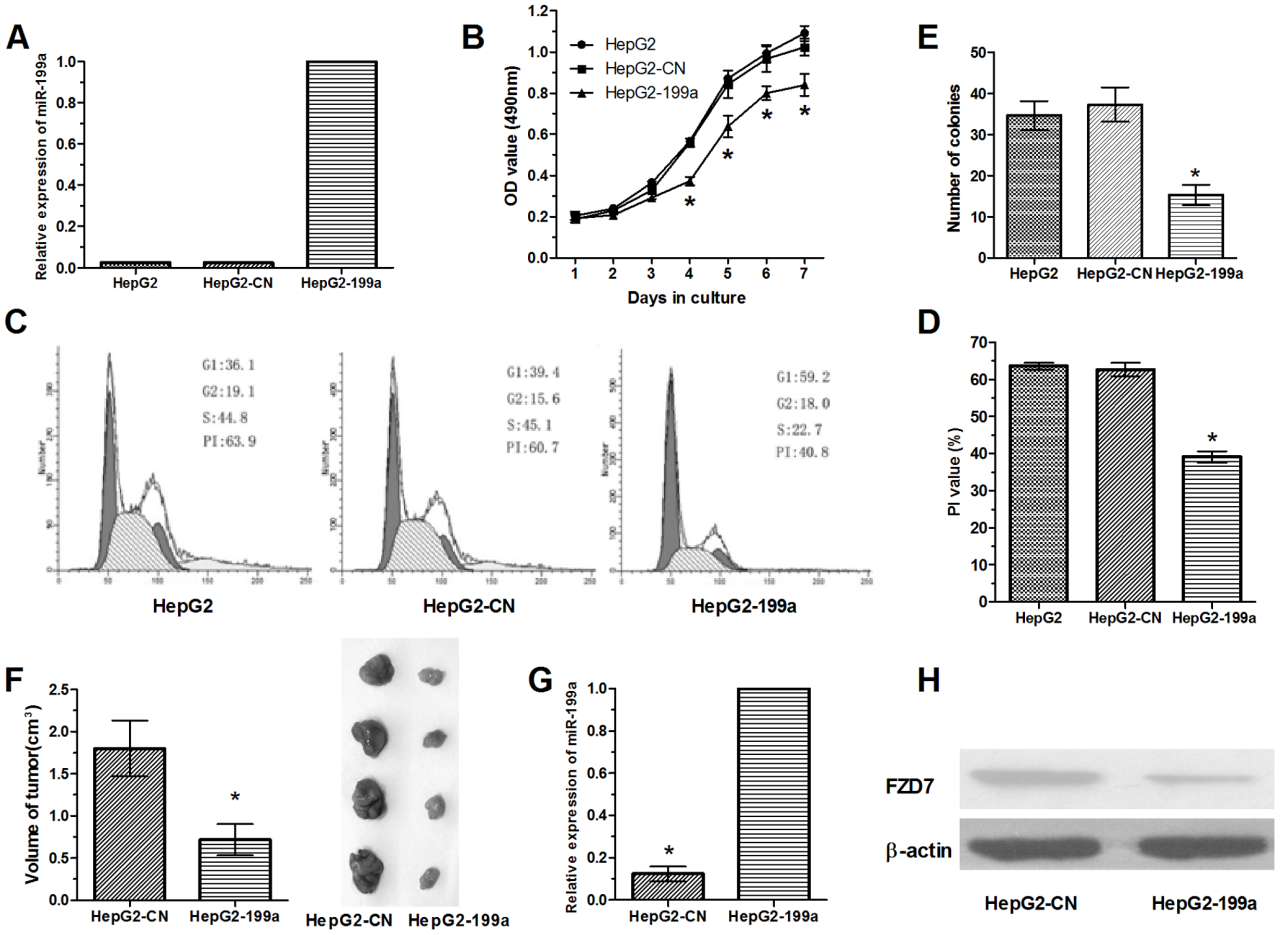
Over-expression of miR-199a represses growth and tumorigenicity *in vitro* and *in vivo*. (A) qRT-PCR analysis confirmed that miR-199a expression was significantly up-regulated in HepG2-199a cells compared with matched controls. U6 snRNA was used as the internal control. (B) The growth curves are plotted based on the MTT assay results. The value shown is the mean of three experiments. *Statistical significance. (C) Cell cycle distribution of HepG2, HepG2-CN, and HepG2-199a cells by flow cytometry. (D) The proliferation index (PI) of HepG2, HepG2-CN, and HepG2-199a cells, as determined by flow cytometry. The PI values that are shown are the mean of three repetitions and are expressed as the mean ± SD. (E) Colony numbers of HepG2-CN and HepG2-199a cells in soft agar. Each soft agar assay was performed in triplicate, and the results are expressed as the mean number of colonies ± SD. (F) Tumor size of HepG2-CN and HepG2-199a cells in nude mice. (G) MiR-199a expression in whole tumor tissue extracts by qRT-PCR analysis. (H) FZD7 expression in whole tumor tissue extracts by Western blot analysis.

To determine whether miR-199a can inhibit the proliferation of HCC cells, lentivirus-mediated miR-199a was transfected into HepG2 cells (HepG2-199a). As observed in Figure 3A, the expression of miR-199a was markedly up-regulated in HepG2-199a cells compared with those in the controls. MTT assays showed that HepG2 cells with increased miR-199a expression (HepG2-199a) proliferated at a slower rate than did control cells, and statistical analysis showed a significant difference after culture for four days (Figure 3B). The cell cycle of these cells was then measured by flow cytometry. The results indicated that 44.8% of HepG2 cells and 45.1% of HepG2-CN cells were in S-phase, whereas 22.7% of HepG2-199a cells were in S-phase (P<0.01; Figure 3C and Figure 3D), suggesting that miR-199a represses the entry of cells into S-phase. Together, the results show that miR-199a can repress the cell cycle and therefore inhibit the proliferation of HepG2 cells.

Then, colony formation assays of parental and transfected cells were evaluated by determining the plating efficiency in soft agar. As shown in Figure 3E, HepG2 and HepG2-CN cells yielded 34.67±3.51 and 37.33±4.16 colonies, whereas HepG2-199a cells yielded 15.33±2.52 colonies after three weeks (P<0.01). Hence, the results showed that there was a marked reduction in anchorage-independent growth among cells with up-regulated expression of miR-199a compared with the controls.

Furthermore, the repression potential of miR-199a on the growth of HepG2 cells in nude mice was also determined. The result showed that tumor size was dramatically smaller in HepG2-199a cells than in control cells (P<0.05; Figure 3F), suggesting that restoration of miR-199a expression was directly involved in the inhibition of tumor growth in nude mice. In addition, the total RNA and protein of each representative tumor from the mice were used to analyze the expression levels of miR-199a and FZD7 by qRT-PCR and western blotting, respectively. The expression level of miR-199a was much higher in recovered tumors formed in nude mice injected with HepG2-199a cells than in those injected with HepG2-CN cells (Figure 3G). Inversely, the expression of FZD7 was significantly lower in tumors with HepG2-199a cells than those with HepG2-CN cells (Figure 3H). In conclusion, the data suggest that miR-199a plays a suppressive role in inhibiting the tumorigenicity of HCC cells *in vitro* and *in vivo*.

### FZD7 is a Novel Direct Target of MiR-199a

**Figure 4:**
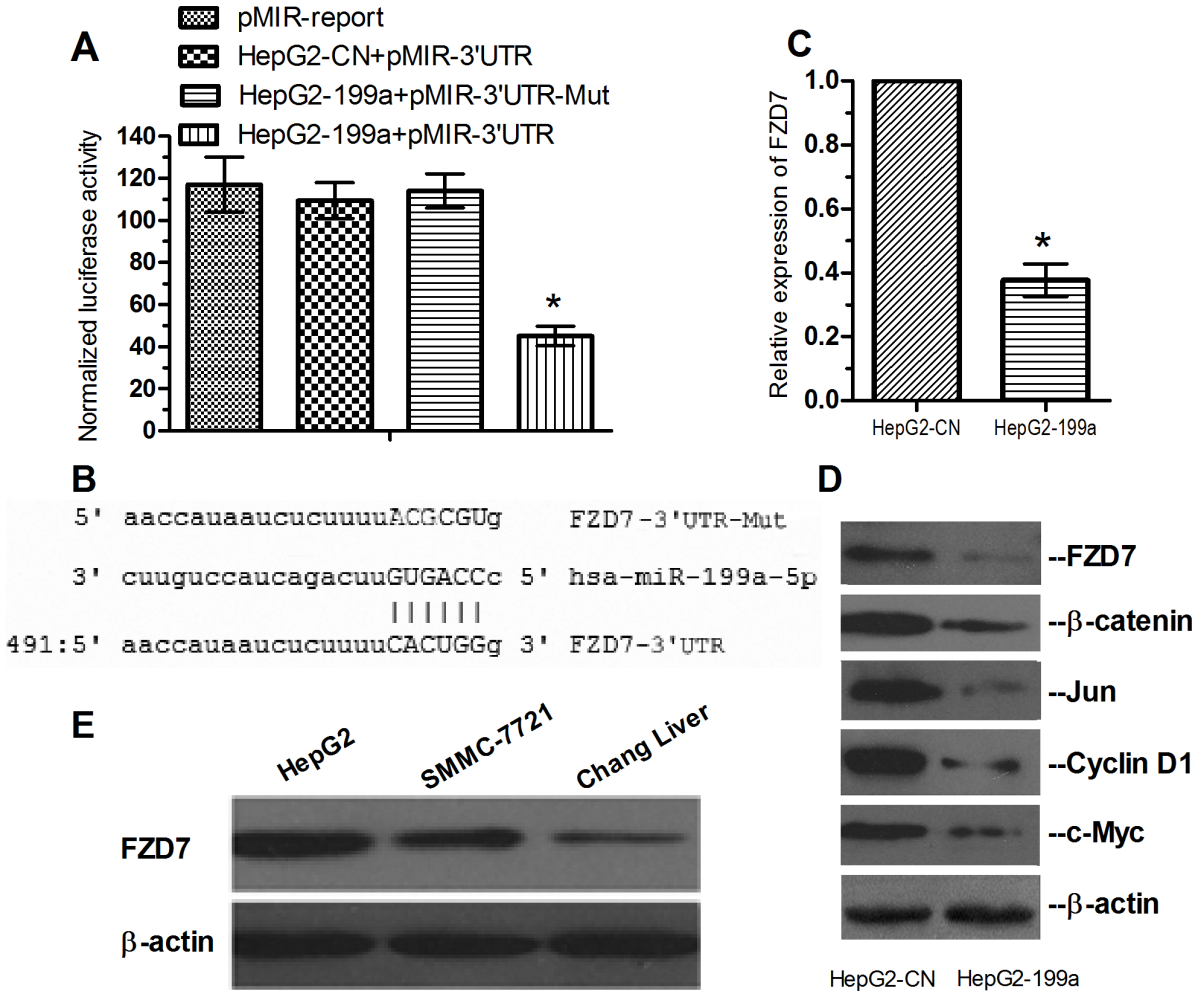
MiR-199a inhibits cell proliferation by directly targeting FZD7. (A) Dual luciferase assays were performed in HepG2-199a and HepG2-NC cells transfected with the firefly luciferase reporter and the control vectors containing Renilla luciferase. The results showed that miR-199a could significantly suppress the luciferase activity of the reporter containing the 3′UTR of FZD7 but had no significant effect on the reporter containing the mutated binding site of FZD7. The data shown are the means ± SD of three independent experiments. (B) The sequences of the miR-199a binding sites within the 3′UTR of FZD7 and the mutated binding site are presented. (C) qRT-PCR analysis showing that the mRNA of FZD7 was significantly decreased in HepG2-199a cells compared with HepG2-NC cells. (D) Western blot analysis confirming that the expression of FZD7 and of its downstream genes was significantly inhibited by miR-199a. β-actin was used as the internal control. (E) The expression of the FZD7 protein in HepG2 and SMMC-7721 cells was higher compared with that in normal Chang liver cells.

To determine the underlying mechanisms by which miR-199a contributes to the progression of HCC, we integrated bioinformatic algorithms, including miRanda, PicTar and TargetScan, to predict the potential target genes of miR-199a. According to the prediction analysis, FZD7, a HCC tumorigenicity-related gene [23], [24], has a putative miR-199a-binding site that maps to the 3′-UTR and is thus of particular interest. To validate the miRNA-target interactions, we constructed luciferase reporters carrying the FZD7 3′-UTR containing the putative miR-199a binding site. As shown in Figure 4A, luciferase assays indicated that the 3′-UTR of FZD7 caused a significant reduction in luciferase activity. However, the variations in the luciferase activity disappeared upon mutation of the key seed region in the 3′UTR of FZD7. The miR-199a binding site in the 3′UTR of FZD7 and the mutated binding site are shown in Figure 4B. The qRT-PCR analysis showed that over-expression of miR-199a significantly repressed the expression of the FZD7 mRNA (Figure 4C). These findings were further verified by the results of the western blot analyses, which revealed that FZD7 expression in HepG2 cells was markedly inhibited by over-expression of miR-199a (Figure 4D). Taken together, these results suggested that miR-199a could significantly suppress the expression of FZD7 through targeting the 3′UTR of its mRNA.

To determine whether miR-199a can inhibit FZD7 expression in clinical HCC tissues. qRT-PCR was performed to measure the expression of FZD7 in HCC samples from 40 patients. As observed in Table 5, Spearman's rank test showed that a significant negative correlation was found between miR-199a and FZD7 expression (r = −0.40, P<0.02). Furthermore, FZD7, as a molecule of the Wnt signaling pathway, and its downstream genes were detected using western blotting. The results indicated that the expression of the FZD7 protein in HCC cell lines was higher compared with that in the normal Chang liver cell line (Figure 4E). In addition, as shown in Figure 4D, over-expression of miR-199a could significantly down-regulate the expression of FZD7 downstream genes, including β-catenin, Jun, Cyclin D1 and Myc. In conclusion, these results indicated that miR-199a represses the development of HCC partly through inhibiting the FZD7 pathway.

**Table 5:** The correlations of miR-199a with FZD7 expression in HCC tissues.

		FZD7 expression			
		Low	High	R value	*P* value
miR-199a	Low	6	14	-0.40	<0.02
expression	High	14	6		

Patients were split into high- and low-expression groups based on the median for miR-199a and FZD7 expression. R: correlation coefficient as evaluated by Spearman's rank correlation coefficient. *P*<0.05 was considered statistically significant.

## Discussion

A growing number of reports have suggested that miRNAs are important regulators of multiple cellular processes and that miRNAs are expressed aberrantly in different types of cancer, including HCC [25], [26]. Studies have revealed that several miRNAs were frequently deregulated in HCC, and some specific miRNAs were found to be associated with the development and progression of HCC [11], [18], [27], [28], [29]. However, the roles of miRNAs in the molecular pathogenesis of HCC are still largely unknown because one miRNA may regulate scores of target genes and a single mRNA may be regulated by multiple miRNAs [30], all of which might function alone or in a cooperative manner. Thus, exploring and understanding the more aberrantly expressed miRNAs may help to better reveal the mechanisms underlying HCC carcinogenesis and progression.

MiR-199a is located on chromosome 19 within intron 14 of the dynamin-2 gene. Previous studies showed that miR-199a expression was diversely deregulated in several types of cancer, including HCC. For instance, miR-199a was found to be down-regulated in ovarian cancer [31], renal cancer [32], prostate cancer [33], [34], colon cancer, bladder cancer [33] and oral squamous cell carcinoma [35], but it was up-regulated in cervical carcinoma [36], gastric cancer [37], [38] and bronchial squamous cell carcinoma [39]. The results of the present study are in line with those of the previous study, which showed that miR-199a expression was frequently down-regulated in HCC tissues compared with matched adjacent nonneoplastic tissues. This finding coincides with our *in vitro* observations that miR-199a is down-regulated in HCC cell lines compared with a normal hepatocyte cell line (Chang liver cells). In addition, lower expression of miR-199a was significantly correlated with the malignant potential and poor prognosis of human HCC. Based on these findings, miR-199a seems to be implicated in HCC development and progression. Lentiviral vectors encoding miRNAs are useful laboratory tools to study gene function. Lentiviral vectors provide efficient gene delivery *in vitro* and can infect nondividing cells. To explore the functions of miR-199a in HCC, HepG2 cells with lower endogenous expression of miR-199a were transfected using lentiviral vectors, leading to the forced expression of the miRNA. Our findings demonstrated that over-expression of miR-199a could inhibit the proliferation of HepG2 cells and could repress cell cycle progression by inducing G0/G1 cell cycle arrest. In addition, the results showed that enforced expression of miR-199a in HepG2 cells could repress the anchorage-independent growth of HepG2 cells in soft agar, suggesting that miR-199a might be a tumor suppressor in hepatocarcinogenesis. This finding was further supported by the finding that the over-expression of miR-199a repressed tumor formation and growth in nude mice. In fact, a miRNA is usually down-regulated in a particular human cancer and can have tumor-suppressor-like effects if the main targets for that specific cell type are oncogenes.

It is generally accepted that miRNAs exert their function through regulating the expression of their downstream target genes. We integrated bioinformatic algorithms, including miRanda, PicTar and TargetScan, to identify the potential target genes of miR-199a. Among the potential mRNAs targeted by miR-199a, FZD7 was particularly interesting. Previous studies indicated that a functional interaction between FZD7 and Wnt3 leads to activation of the Wnt/β-catenin signaling pathway in HCC cells and may play an important role in hepatocarcinogenesis [23]. Among the FZD family, FZD7 appears to be the most important Wnt receptor involved in cancer development and progression, and FZD7 is most commonly up-regulated in a variety of cancers, including colorectal cancer [40], HCC [24], esophageal cancer [41], breast cancer [42], lung cancer [43], Wilm's tumor [44], gastric cancer [45] and melanoma [43]. The over-activation of Wnt signaling with the up-regulated expression of FZD7 in various types of cancer and the roles of FZD7 in cancer stem cell biology suggest that FZD7 might serve as a therapeutic target for certain cancers [46]. Several research groups have attenuated the action of over-expressed Fzd7 in cancer cells using different methods such as an anti-FZD7 antibody, an extracellular peptide of FZD7 (soluble FZD7 peptide), small interfering peptides or a small molecule inhibitor [40], [47], [48], [49], [50], [51]. Therefore, targeted inhibition of FZD7 represents a rational and promising new approach for cancer therapy. To experimentally validate this computer prediction, luciferase reporter assays confirmed that FZD7 was a target gene of miR-199a. These data were further strengthened by results from exploring the protein levels of FZD7 in HepG2 cells by western blotting, which showed that the over-expression of miR-199a markedly decreased FZD7 protein expression. Then, the downstream genes of FZD7, including β-catenin, Jun, Cyclin D1 and Myc were investigated using western blot analysis, and the results demonstrated that over-expression of miR-199a could significantly down-regulate the expression of the downstream genes of FZD7. Moreover, the co-expression of miR-199a and its target gene FZD7 were detected in HCC tissues. The results showed that miR-199a was inversely correlated with FZD7 expression in HCC tissues. Taken together, these results strongly suggested that miR-199a might function as a tumor suppressor partly by mediating the repression of FZD7 expression in HCC development.

In conclusion, the data presented here strongly indicate that miR-199a acts as a tumor suppressor in HCC. Our present study showed that miR-199a is frequently down-regulated and inversely correlated with poor prognosis in HCC patients. In addition, restoration of miR-199a expression in HCC cells leads to inhibition of the cell proliferation and of the cell cycle partly through down-regulating FZD7 *in vitro* and *in vivo*. These findings not only help us to better elucidate the molecular mechanisms of hepatocarcinogenesis from a fresh perspective but also provide a new theoretical basis to further investigate miR-199a as a potential biomarker and a promising approach for HCC treatment.

## Supporting Information

Table S1 The relative expression of miR-199a in all HCC tissues.
(DOC)
