# Supplementary material for: MiR-199a Regulates Cell Proliferation and Survival by Targeting FZD7
Source: PLoS One. 2014 Oct 14;9(10):e110074. doi: 10.1371/journal.pone.0110074 (PMC4196968; doi:10.1371/journal.pone.0110074)
Supplement: Table S1 — The relative expression of miR-199a in all HCC tissues. (DOC) [file pone.0110074.s001.doc]

**Table S1.** The relative expression of miR-199a in all HCC tissues

| Sample No. | Relative expression of miR-199a | Sample No. | Relative expression of miR-199a |
| --- | --- | --- | --- |
| 1 | 3.9 | 21 | 1.3 |
| 2 | 1.9 | 22 | 0.8 |
| 3 | 2.7 | 23 | 1.2 |
| 4 | 1.5 | 24 | 1.0 |
| 5 | 4.8 | 25 | 3.9 |
| 6 | 4.3 | 26 | 0.7 |
| 7 | 6.1 | 27 | 2.1 |
| 8 | 4.1 | 28 | 1.8 |
| 9 | 3.9 | 29 | 1.9 |
| 10 | 4.9 | 30 | 5.3 |
| 11 | 2.4 | 31 | 3.2 |
| 12 | 3.1 | 32 | 2.1 |
| 13 | 3.7 | 33 | 5.2 |
| 14 | 4.3 | 34 | 2.0 |
| 15 | 2.7 | 35 | 1.5 |
| 16 | 2.4 | 36 | 0.9 |
| 17 | 3.3 | 37 | 5.2 |
| 18 | 2.9 | 38 | 2.7 |
| 19 | 2.1 | 39 | 4.9 |
| 20 | 2.9 | 40 | 7.3 |
